# Single-session robot-assisted removal of a retained ureteral stent with complex proximal and distal encrustation: A case report

**DOI:** 10.1177/17562872261485779

**Published:** 2026-09-02

**Authors:** Maximo Alvarez, Cole Freedman, Emma Romero, Paul Kim, Alexis Garza, Harras Zaid

**Affiliations:** 1 441554 The University of Texas Rio Grande Valley School of Medicine, Edinburg, TX, USA; 2 Urology Institute at Renaissance, 205980 Doctors Hospital at Renaissance, Edinburg, TX, USA; 3 21976 Department of Surgery and Perioperative Care, Dell Medical School, The University of Texas at Austin, Austin, TX, USA

**Keywords:** forgotten ureteral stent, encrustation, robotic pyelolithotomy, cystolithotomy, FECal algorithm, healthcare disparities, urolithiasis, case report

## Abstract

Retained encrusted ureteral stents represent a complex urologic problem, particularly in patients with limited healthcare access. We present a 28-year-old undocumented female with a 10-month retained ureteral stent who developed massive right-sided encrustation, including a 3 cm proximal renal stone near the renal pelvis and a 5-6 cm bladder stone, classified as FECal Grade IV-V. A single-session combined approach consisting of robot-assisted cystolithotomy, intraoperative ureteroscopy with stent exchange, and robot-assisted pyelolithotomy was performed successfully. Intraoperative ureteroscopy between operative phases confirmed ureteral clearance and enabled immediate stent placement without repositioning delay. This case supports the use of combined robotic approaches for high-grade disease and illustrates how structural barriers drive late presentation and increased surgical complexity.

## Introduction

Ureteral stents play a pivotal role in relieving ureteral obstruction, facilitating instrumentation, and maintaining ureteral patency during healing. Despite their clinical utility, stents left in situ beyond their intended dwell time carry well-recognized consequences, ranging from encrustation and recurrent infection to renal deterioration and life-threatening sepsis. Encrustation, defined as mineral deposition of calcium, oxalate, and phosphorus onto the stent surface, is detectable in 27% of patients at 6 weeks, 57% between 6 and 12 weeks, and 76% beyond 12 weeks.^1,2^ Prolonged indwelling time is the primary and most consistent risk factor for encrustation, while concurrent urease-producing infections, prior stone disease, and limited healthcare access compound encrustation risk and accelerate stone burden progression.^1,3–6^ Management of retained encrusted ureteral stents with large associated stone burden remains surgically challenging, often requiring staged intervention. Standard endoscopic approaches, including percutaneous nephrolithotomy (PCNL), extracorporeal shock wave lithotripsy (ESWL), ureteroscopy (URS), and cystolitholapaxy may be insufficient in cases of severe or multisite encrustation and frequently necessitate more than one operative encounter.^7^

Combined robotic approaches have emerged as a promising single-session alternative; no standardized technique exists for cases involving simultaneous large proximal renal and distal bladder stone burden on a retained stent. We present a case of massive stent encrustation at the proximal and distal ends following a 10-month retention of a ureteral stent in an undocumented patient with insurance-related loss to follow-up, and describe a single-session approach combining robot-assisted cystolithotomy, intraoperative ureteroscopy with stent exchange, and robot-assisted pyelolithotomy. This case report is presented in accordance with the CARE reporting checklist.^8^

## Case presentation

### Patient information and presentation

A 28-year-old undocumented Hispanic female with obesity and recurrent *Proteus mirabilis* urinary tract infections presented to the emergency department at Dell Seton Medical Center at the University of Texas with gross hematuria with clots and worsening bilateral pelvic and flank pain. She is married, has two children, and works in food service. This was her third presentation to an emergency room for the same retained ureteral stent. Prior history was collected from both the patient and her spouse, who was present during the consultation.

### Past medical and surgical history

Past medical history included recurrent *Proteus mirabilis* urinary tract infections and obesity. Her surgical history was limited to placement of a right-sided double-J ureteral stent in February 2025 at another facility, performed for a 5 mm right obstructing distal ureteral stone with complicating urosepsis. The patient reported no prior nephrolithiasis, interventions, or family history.

### Social history and access barriers

The patient resides with her spouse and two children. She is employed in food service and identified herself as an undocumented immigrant without health insurance. Her lack of insurance was the primary barrier to urologic follow-up during her 10-month ureteral stent placement. She denied tobacco, alcohol, or recreational drug use.^9,10^

### Medications and allergies

At the time of consultation, the patient was receiving ceftriaxone 1 g IV q24h for active *Proteus* infection initiated on admission. Scheduled medications included ondansetron, magnesium sulfate, and potassium chloride to correct hypokalemia. PRN medications included morphine, oxycodone, hydrocodone-acetaminophen, bisacodyl, docusate-senna, aluminum hydroxide, and potassium chloride. Home medication included ketorolac 10 mg daily. No allergies or adverse drug reactions were reported.

### Review of systems and clinical examination

At the time of her final presentation, the patient reported gross hematuria with clots and bilateral pelvic and flank pain. She was afebrile with stable vital signs and no systemic signs of sepsis. Preoperative creatinine was 0.6 mg/dL with preserved renal parenchyma on imaging. She presented with mild normocytic anemia (Hgb 11.2 g/dL) and mild hypokalemia (K 3.2 mEq/L), both of which were addressed prior to the procedure.

## Timeline

The clinical timeline is summarized in Table 1.

**Table 1.** The clinical timeline of a retained, encrusted stent requiring operative (OR) and emergency room (ER) interventions over 10 months.

Date	Event
February 10, 2025	Presents to outside facility, CT identifies an obstructing 5 mm distal right ureteral stone with urosepsis secondary to Proteus mirabilis. Right double-J ureteral stent placed. Discharged with outpatient follow-up instructions. Follow-up not completed due to lack of insurance coverage.
May 2025	Second presentation to an outside emergency department with stent-associated pain. CT demonstrates 2-3 cm calcification of the distal stent coil. Urology was not consulted. Patient is unable to access outpatient urologic care due to insurance barriers.
December 25, 2025	Third presentation to Dell Seton Medical Center with gross hematuria and worsening pain, approximately 10 months after initial stent placement. CT demonstrates 3 cm proximal and 5 cm distal encrustation on the retained stent with incidental left renal stones. Initiated on ceftriaxone; hypokalemia corrected. Surgical consent obtained.
December 26, 2025	Single-session robot-assisted cystolithotomy, intraoperative right ureteroscopy with stent exchange, and robot-assisted right pyelolithotomy were performed without intraoperative complications. Estimated blood loss is 100 mL. Replacement 6-French x 26 cm double-J stent placed.

### Diagnostic assessment

**Figure 1.**
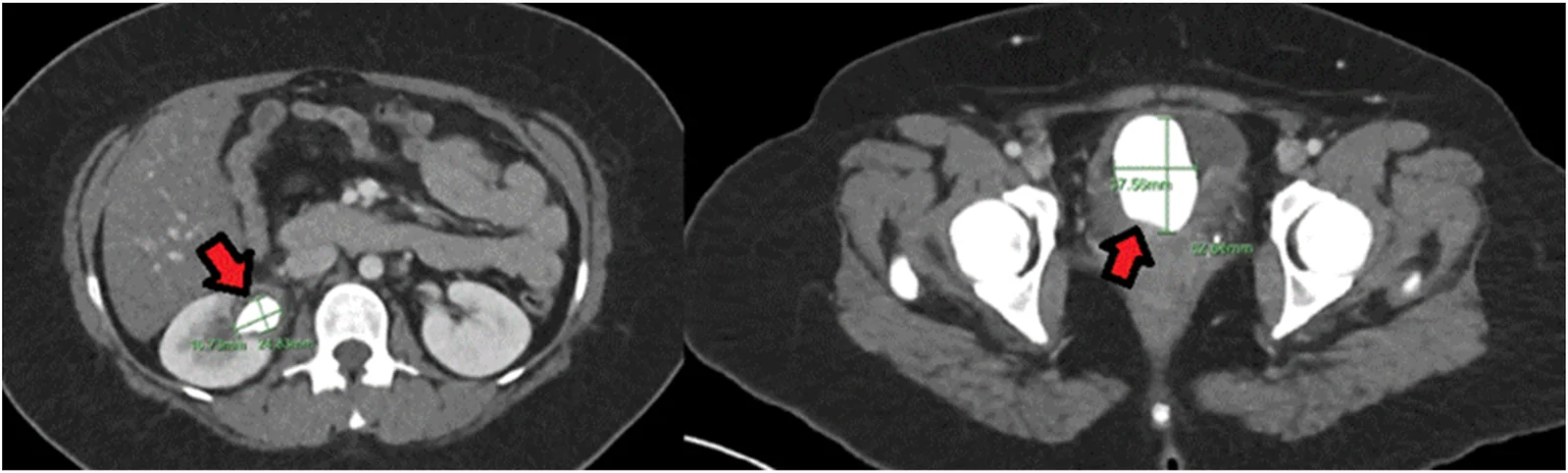
Axial contrast-enhanced CT of the abdomen and pelvis demonstrating a right-sided renal and bladder calculi with a retained ureteral stent.

**Figure 2.**
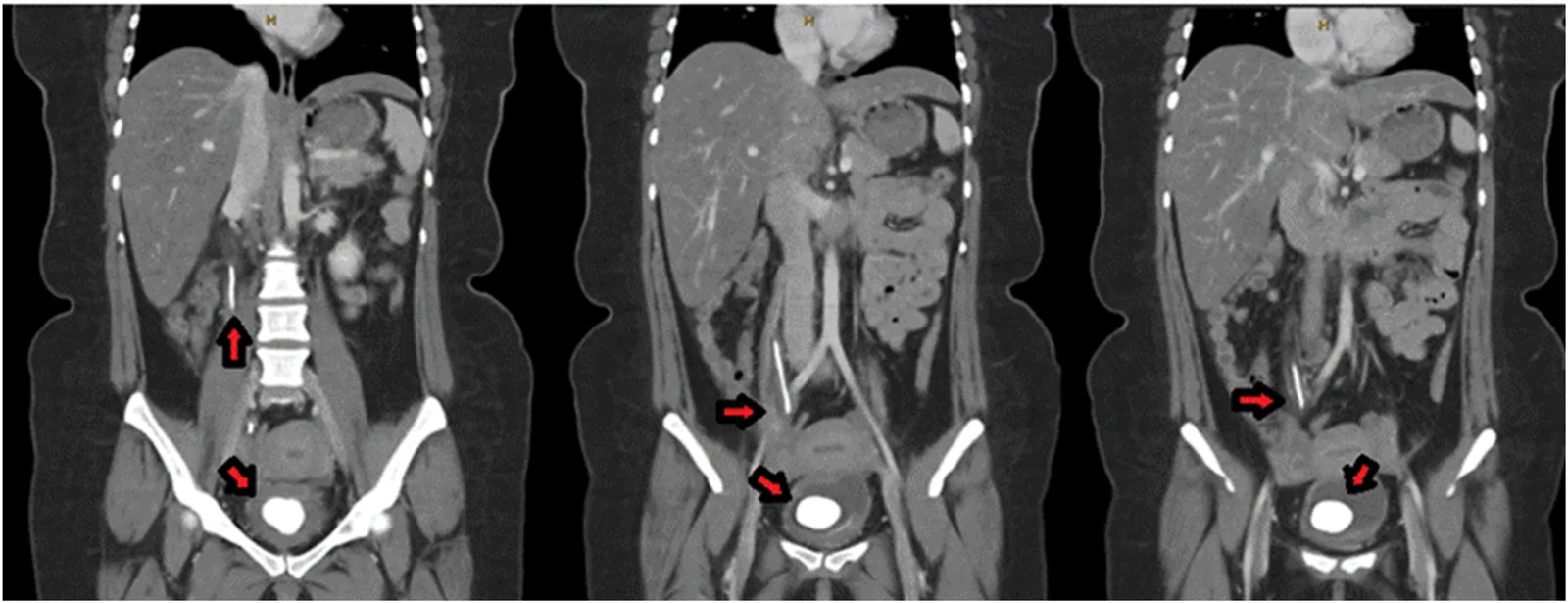
Coronal contrast-enhanced CT of the abdomen and pelvis demonstrating the calcified retained stent and associated distal calculi.

CT abdomen and pelvis with contrast at initial presentation in February 2025 identified a 5 mm obstructing distal right ureteral stone with associated urosepsis. At the May 2025 return visit, CT demonstrated interval development of a 2-3 cm calcification encasing the distal stent coil, representing early-to-moderate distal encrustation. At the final presentation in December 2025, CT demonstrated a 3 cm encrustation encasing the proximal stent coil at the level of the renal pelvis and a 5-6 cm calcification encasing the distal coil within the bladder, visualized in Figure 1. The stent body, proximal coil, and distal coil were all involved, consistent with diffuse encrustation at both ends with intervening ureteral segments as noted in Figure 2. Renal parenchyma appeared preserved bilaterally. Incidental nonobstructive stones were noted in the left kidney. Preoperative laboratory evaluation demonstrated mild normocytic anemia and mild hypokalemia as noted above.

## Intervention

### Preoperative optimization

The patient completed 48 hours of IV ceftriaxone 1 g q24h prior to the procedure to reduce infectious risk. Mild hypokalemia (K 3.2 mEq/L) was corrected with IV and oral potassium chloride supplementation. Normocytic anemia (Hgb 11.2 g/dL) was deemed acceptable for the planned procedure. No formal bowel prep was initiated. The patient was kept nil per os prior to the procedure.

### Operative approach

A single-session combined robotic and endoscopic approach was selected over staged endoscopy given the size of the distal calcification, the patient’s obesity, and the risk of incomplete clearance across multiple anesthetic encounters. These clinical factors drove the operative decision independent of the patient’s insurance status, which accounts for the severity of disease at presentation rather than the choice of technique. The procedure was performed using the Intuitive Surgical da Vinci Surgical System.

### Operative details

The procedure consisted of three sequential phases performed under general anesthesia.

#### Phase 1 — Robot-assisted cystolithotomy

**Figure 3.**
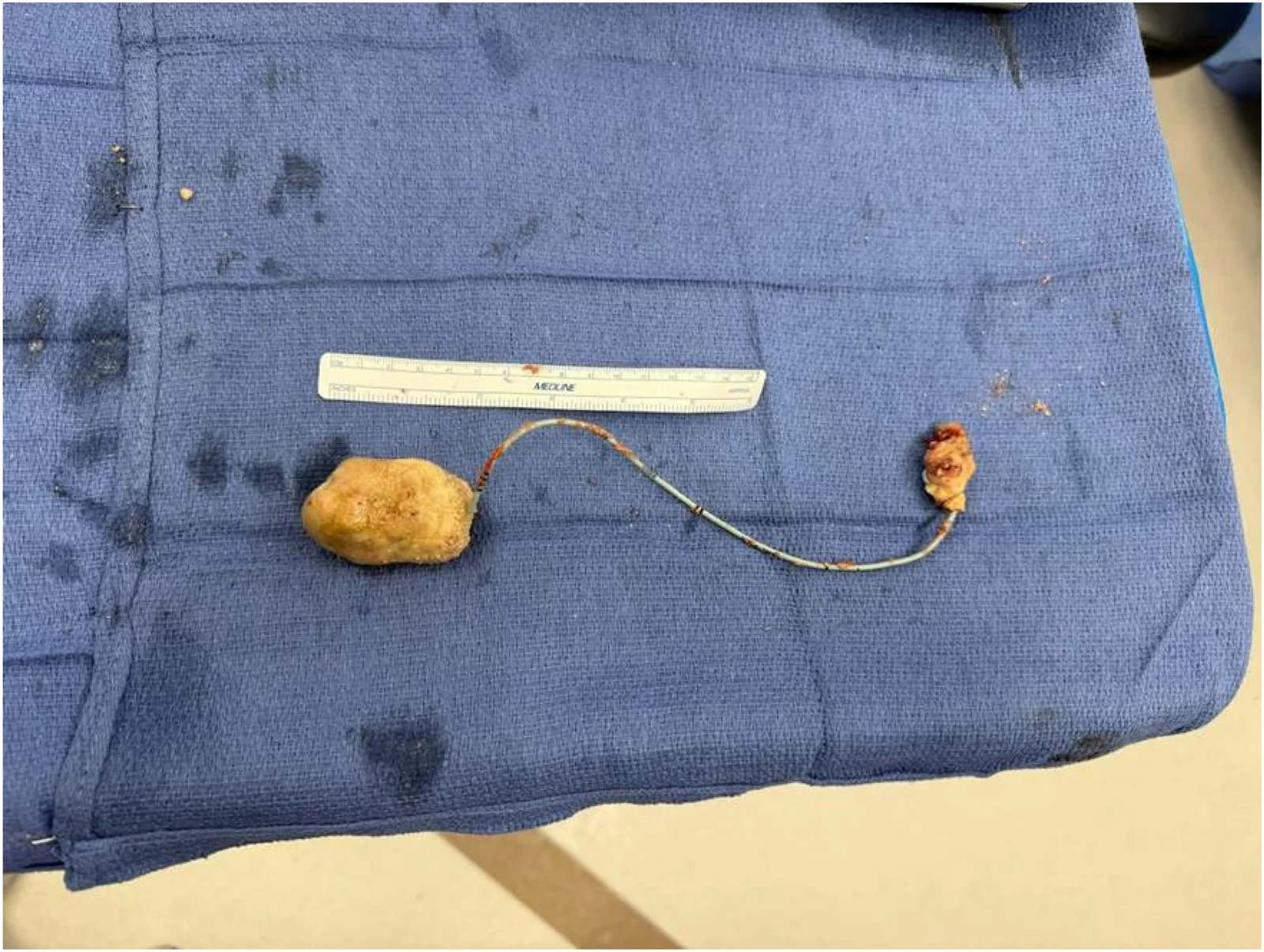
Inspection of surgically removed ureteral stent with attached calculi after complete removal across both operative phases.

The patient was positioned in lithotomy, and the robot was docked targeting the pelvis. Pneumoperitoneum was established, and a midline cystotomy at the bladder dome was performed. The 6 cm calcified distal coil was grasped, the distal stent was divided at a stone-free segment, and the calcified distal coil was removed and bagged. The proximal stent fragment and its associated renal calcification were intentionally left in situ at this stage. Figure 3, in which both calcified ends appear connected by the stent body, reflects inspection after complete removal across both phases, and not a single intact piece at extraction. Significant cystitis and mucosal edema were noted. The cystotomy was closed in two layers with 3-0 V-Loc, and an intraoperative bladder distention confirmed no leakage at 300 mL.

#### Phase 2 — Intraoperative ureteroscopy with stent exchange

Ureteroscopy was performed with the semi-rigid ureteroscope, advanced to the ureteropelvic junction alongside the retained proximal stent fragment. No intraureteral stone burden was identified. A 6-French x 26 cm double-J ureteral stent was placed over a second wire, with coil positioning confirmed under fluoroscopy. This ureteroscopic interphase, performed prior to patient repositioning between the pelvic and retroperitoneal phases, confirmed ureteral clearance, secured drainage before the pyelotomy, and eliminated the need for a separate ureteroscopic encounter. This stepwise approach has not been consistently described in prior combined robotic series and represents a reproducible technical refinement for cases of similarly complex multi-site stone burden.

#### Phase 3 — Robot-assisted right pyelolithotomy

The patient was repositioned to the right flank position via a transperitoneal approach. The ureter was traced to the collecting system,m with periureteral edema not consistent with chronic inflammation. A longitudinal pyelotomy was performed, and the 3 cm proximal calcification with the retained proximal stent fragment was removed in its entirety. Both specimens were placed in an 8mm endocatch bag and extracted from the umbilical assistant port at the end of the case. The collecting system was closed with running 3-0 V-Loc; hemostasis was achieved with Vistaseal fibrin sealant and Arista hemostat. Jackson-Pratt drains were placed in the pelvis and renal fossa. Estimated blood loss was 100 mL with no intraoperative complications.

Suture materials included 3-0 V-Loc for bladder and collecting system closure and Vicryl UR6 for fascial closure.

### Operator and setting

The procedure was performed by Harras Zaid, MD, a board-certified urologist and fellowship-trained urologic oncologist at Dell Seton Medical Center at the University of Texas at Austin, an academic medical center with extensive robotic surgical capabilities. Dr. Zaid completed medical training at the University of California, San Francisco, urology residency at Vanderbilt University Medical Center, and fellowship training in urologic oncology at the Mayo Clinic. He is currently an assistant professor in the Department of Surgery and Perioperative Care at Dell Medical School.

### Deviations from planned management

No significant deviations from the planned procedure occurred. The decision to withhold intraoperative pyeloscopy was made based on the grossly normal appearance of the collecting system and positive visualization of the replacement stent. This represents a minor deviation judged to carry acceptable risk.

### Follow-up and outcomes

The patient had a foley for 10 days, which was removed from the clinic. Additionally, her new stent was attached to a string and to be removed for 4-6 weeks post-operatively. No perioperative laboratory assessments are available. The patient was seen postoperatively 5 weeks later, her stent was removed, and she remained asymptomatic. The recovery period and stent removal were tolerated well. Unfortunately, she was once again lost to follow-up after stent removal. Patient-reported measures are accordingly not available.

## Discussion

### Pathophysiology and risk factors

Encrustation of indwelling ureteral stents results from mineral deposition on the luminal and external stent surfaces, compromising tensile strength and raising the risk of stent fracture or ureteral avulsion during removal. Indwelling time is the dominant risk factor; encrustation rates rise sharply beyond 45 days and reach 76% after 12 weeks of dwell.^2,3^ Bacterial colonization and biofilm formation compound encrustation risk by providing a scaffold for crystal deposition, with colonization approaching 82% at 90-120 days.^11–13^ Beyond encrustation, retained stents carry a well-documented burden of urinary symptoms, including frequency, dysuria, hematuria, and pelvic pain, which compound the clinical consequences of delayed removal and represent an independent reason for timely stent management.^14^ In this patient, a 10-month dwell time combined with recurrent urease-producing *Proteus* infections created conditions for exceptional stone burden on both ends of the stent.^15–17^

### Structural barriers and social context

Socioeconomic and structural barriers, though not independent predictors of encrustation, contribute indirectly by increasing the likelihood of delayed stent removal and loss to follow-up.^5,6^ An estimated 10.5 million undocumented immigrants reside in the United States, and this population disproportionately faces financial, geographic, and language barriers to primary care.^18^ As a result, emergency services often substitute for longitudinal care,^18,19^ and outpatient procedures requiring prepayment remain inaccessible. In the setting of emergently placed ureteral stents, this pattern systematically converts a manageable outpatient procedure into a staged operative problem. Policy interventions directed at insurance access and primary care infrastructure for this population may reduce the frequency and severity of presentations like this one.^20–22^

### Operative decision-making and the FECal algorithm

**Figure 4.**
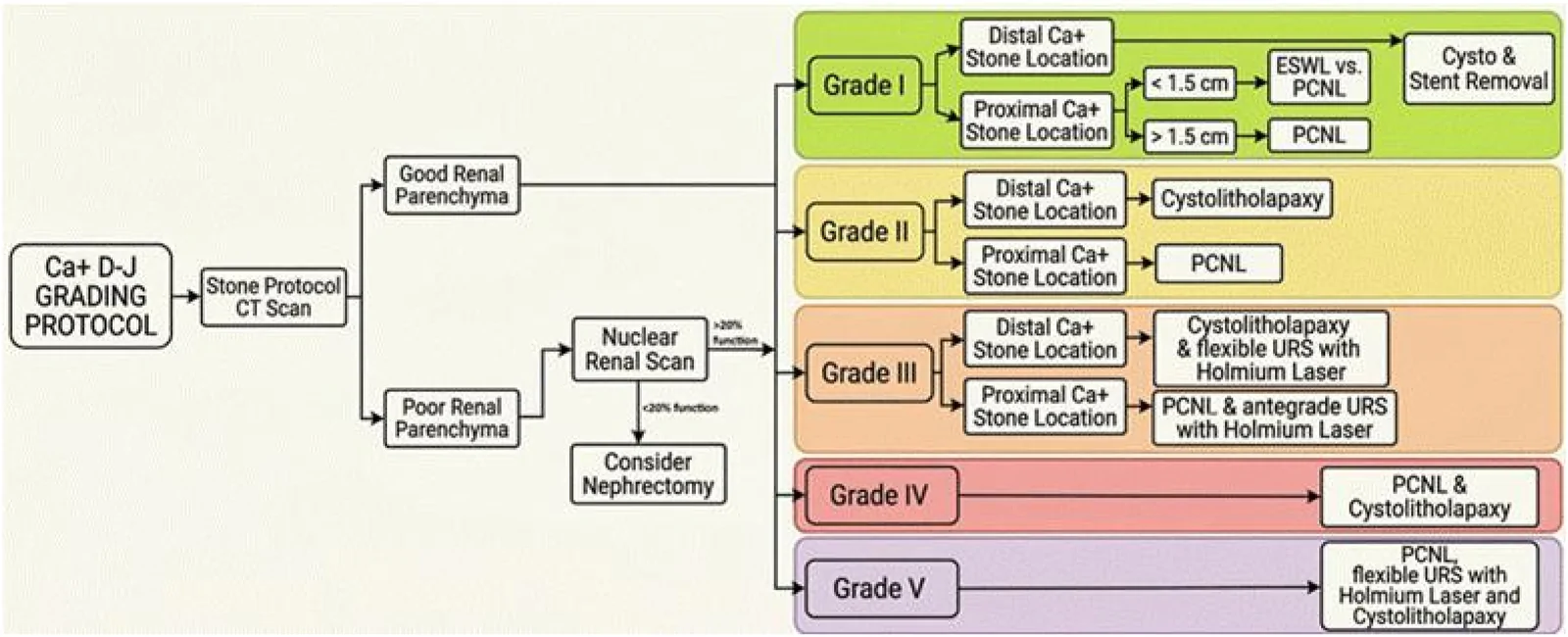
Original depiction of FECAL grading algorithm for management of retained encrusted ureteral stents.

The forgotten, encrusted, calcified (FECal) algorithm guides management of retained ureteral stents according to the location and severity of encrustation across five grades.^7^ Grade I involves minimal linear encrustation of one coil; Grade V represents diffuse, bulky encrustation of both coils and the intervening ureteral segment. The grading algorithm is best visualized in Figure 4. Based on CT findings demonstrating complete encrustation of both coils with a 3 cm proximal renal stone and a 5-6 cm bladder stone, this patient was classified as FECAL Grade IV-V, for which standard management consists of PCNL combined with cystolitholapaxy.^7^ Broader nomogram frameworks, including those described by the TOWER Endourological Society Research Group, further contextualize the role of predictive scoring in endourologic decision-making and may complement the FECAL algorithm in future risk stratification efforts for complex retained stent cases.^23^

Standard FECal Grade IV-V management was deemed insufficient for this presentation. The 5-6 cm distal calcification exceeded what endoscopic cystolitholapaxy could reasonably clear in a single session without substantial risk of incomplete fragmentation and postoperative hematuria. A staged approach would have required at least two separate anesthetic events in a patient with obesity and recurrent infections, compounding perioperative risk. A single-session combined robotic approach was therefore selected, building on published combined robotic techniques.^24^ The operative decision was driven primarily by the patient’s structural barriers, specifically the need to ensure complete removal of the proximal and distal stones, given the high likelihood of loss to follow up with the retained stent fragment. Nonetheless, this approach would also be warranted in a fully insured patient. The structural barriers this patient experiences are responsible for the development and severity of her encrusted stents, but not the choice of operative technique. A similarly complex stone burden in a fully insured patient would justify the same approach on clinical grounds alone, though such a presentation would be unlikely in the absence of the access barriers.

### Technical innovation: The ureteroscopic interphase

The defining technical feature of this case was the addition of intraoperative ureteroscopy between the pelvic and retroperitoneal phases. After cystolithotomy and distal stent extraction, ureteroscopy was performed prior to patient repositioning. This confirmed the absence of intraureteral stone burden and enabled immediate placement of a new double-J stent under fluoroscopic guidance, securing ureteral drainage before the pyelotomy phase. This sequence eliminated the need for a separate ureteroscopic procedure and avoided the risk of operating into an unsecured or obstructed collecting system. Prior combined robotic series, including Aron et al., describe multimodal approaches to difficult stent removal but do not consistently incorporate this ureteroscopic interphase between operative phases.^24^ This stepwise refinement represents a reproducible technical contribution applicable to similarly complex multi-site presentations, though its generalizability is limited by this single case design.

## Limitations

Several limitations of this report warrant acknowledgment. The most consequential is the absence of postoperative laboratory data, including renal function, urine culture, and inflammatory markers, which limits objective assessment of perioperative recovery. While the patient was seen at approximately five weeks postoperatively and remained asymptomatic at stent removal, she was subsequently lost to follow-up. This development does not allow for the evaluation of renal outcomes or delayed complications. This limits the ability to draw definitive conclusions about the durability of the approach. Additionally, the patient’s incidental contralateral renal stones were not addressed, and their subsequent management is unknown.

As an inherent feature of the single case design, this report cannot establish generalizable conclusions regarding optimal management of FECAL Grade IV-V disease. While the technical refinements involving the intraoperative ureteroscopic interphase appear reproducible, their benefit over alternative sequencing strategies cannot be formally quantified from a single experience. Prospective series data would be necessary to validate these observations across a broader patient population.

## Conclusion

Retained ureteral stents with severe encrustation represent a challenging clinical problem, particularly in patients with prolonged indwelling time and limited access to follow-up care. This case demonstrates that a combined robotic and endoscopic approach is an effective strategy for managing complex, multi-site stone burden involving both the kidney and bladder. In patients with FECAL Grade IV-V disease, where standard endoscopic management carries substantial risk of incomplete clearance and staged intervention compounds anesthetic exposure, a single-session robotic approach offers a meaningful alternative. The intraoperative ureteroscopy interphase between the pelvic and retroperitoneal phases allowed ureteral clearance confirmation and immediate stent exchange without repositioning-related delay, a technical step not consistently described in prior combined robotic series. Beyond the technical dimensions, this case underscores the role of structural barriers, including undocumented immigration status, lack of insurance, and fragmented follow-up infrastructure, in driving delayed presentation and compounding surgical complexity. Early identification of at-risk patients alongside systemic investment in primary care access may represent the most consequential intervention available to prevent the degree of encrustation and downstream morbidity seen here.

## Supplemental material

Supplemental material - Single-session robot-assisted removal of a retained ureteral stent with complex proximal and distal encrustation: A case report Supplemental material for Single-session robot-assisted removal of a retained ureteral stent with complex proximal and distal encrustation: A case report by Maximo Alvarez, Cole Freedman, Emma Romero, Paul Kim, Alexis Garza, Harras Zaid in Therapeutic Advances in Urology.

## Data Availability

Data sharing is not applicable to this article as no datasets were generated or analyzed.*
