# Supplementary material for: Single-session robot-assisted removal of a retained ureteral stent with complex proximal and distal encrustation: A case report
Source: Ther Adv Urol. 2026 Sep 2;18:17562872261485779. doi: 10.1177/17562872261485779 (PMC13539040; doi:10.1177/17562872261485779)
Supplement: Supplementary file 1 [file sj-pdf-1-tau-10.1177_17562872261485779.pdf]

CARE Checklist			
**Topic**	**Item**	**Checklist item description**	**Page Number**
**Title**	**1**	The diagnosis or intervention of primary focus followed by the words "case report".	1
**Key Words**	**2**	2 to 5 key words that identify diagnoses or interventions in this case report (including "case report").	1
**Abstract**	**3a**	Introduction — What is unique about this case and what does it add to the scientific literature?	1
	**3b**	The patient's main concerns and important clinical findings.	
	**3c**	The primary diagnoses, interventions, and outcomes.	
	**3d**	Conclusion — What are one or more "take-away" lessons from this case report?	
**Introduction**	**4**	Briefly summarizes why this case is unique and may include medical literature references.	1-2
**Patient Information**	**5a**	De-identified patient specific information.	2
	**5b**	Primary concerns and symptoms of the patient.	
	**5c**	Medical, family, and psychosocial history including relevant genetic information.	
	**5d**	Relevant past interventions and their outcomes.	
**Clinical Findings**	**6**	Describe significant physical examination (PE) and important clinical findings.	3
**Timeline**	**7**	Historical and current information from this episode of care organized as a timeline (figure or table).	3, 9
**Diagnostic Assessment**	**8a**	Diagnostic methods (PE, laboratory testing, imaging, surveys).	1, 3, 6
	**8b**	Diagnostic challenges.	
	**8c**	Diagnosis (including other diagnoses considered).	
	**8d**	Prognostic characteristics when applicable.	
**Therapeutic Intervention**	**9a**	Types of therapeutic intervention (pharmacologic, surgical, preventive).	3-5
	**9b**	Administration of therapeutic intervention (dosage, strength, duration).	
	**9c**	Changes in therapeutic interventions with explanations.	
**Follow-up and Outcomes**	**10a**	Clinician- and patient-assessed outcomes if available.	4-5
	**10b**	Important follow-up diagnostic and other test results.	
	**10c**	Intervention adherence and tolerability. (How was this assessed?)	
	**10d**	Adverse and unanticipated events.	
**Discussion**	**11a**	Strengths and limitations in your approach to this case.	5-8
	**11b**	Discussion of the relevant medical literature.	
	**11c**	The rationale for your conclusions.	
	**11d**	The primary "take-away" lessons from this case report (without references) in a one paragraph conclusion.	
**Patient Perspective**	**12**	The patient should share their perspective on the treatment(s) they received.	Not included
**Informed Consent**	**13**	The patient should give informed consent. (Provide if requested.)	8
